# Association of Anthropometric Measurements With Lipid Profile in Hypertension

**DOI:** 10.7759/cureus.93080

**Published:** 2025-09-23

**Authors:** Charu Lakhi, Savita Kumari

**Affiliations:** 1 Internal Medicine, Maharishi Markandeshwar Institute of Medical Sciences and Research, Mullana, IND

**Keywords:** cholesterol, dyslipidemia, hypertension, ldl, skinfold thickness, triglycerides, waist circumference

## Abstract

Objectives: To assess whether anthropometric indices (body mass index (BMI), waist circumference, and skinfold thickness) are associated with lipid profile parameters in participants with and without hypertension.

Methods: A case-control study was conducted on 200 participants (100 with hypertension and 100 without hypertension) at Maharishi Markandeshwar Institute of Medical Sciences and Research (MMIMSR), Ambala. Anthropometric parameters included BMI, waist and hip circumference, waist-to-hip ratio, and skinfold thickness (biceps, triceps, subscapular, and suprailiac). Fasting lipid profiles (total cholesterol, low-density lipoproteins cholesterol (LDL-C), high-density lipoproteins cholesterol (HDL-C), and triglycerides) were analyzed using standard enzymatic methods. Statistical analysis was done using SPSS v25.0 with p < 0.05 as significant.

Results: Participants with hypertension had significantly higher BMI, waist and hip circumference, waist-to-hip ratio, and skinfold thickness at triceps, subscapular, and suprailiac sites (p < 0.05). Biceps thickness did not differ significantly (p = 0.174). Total cholesterol, LDL-C, and triglycerides were significantly elevated in participants with hypertension, while HDL-C was lower (p < 0.0001 for all). BMI positively correlated with total cholesterol (r = 0.45), LDL-C (r = 0.42), and triglycerides (r = 0.39) and negatively with HDL-C (r = -0.40), all p < 0.001.

Conclusion: Central obesity was significantly associated with adverse lipid profiles in this sample of hypertensives. Regular assessment of anthropometric and lipid parameters is crucial for early identification and management of cardiometabolic risk.

## Introduction

Hypertension is a major global health concern, affecting approximately 1.28 billion people aged 30-79, with 66.6% residing in developing countries [1]. It arises from a complex interplay of genetic and environmental factors [2]. Age-related arterial changes, such as hardening, atherosclerosis, and vascular collagen disturbances, contribute significantly. Dysregulation of the renin-angiotensin-aldosterone system and neurohumoral pathways can elevate blood pressure and cause organ damage [3]. Obesity and dyslipidemia are key modifiable risk factors, and lifestyle interventions are crucial in prevention and management [4].

Obesity is closely linked with both hypertension and dyslipidemia [5]. Increased abdominal or subcutaneous fat burdens the heart, exacerbating hypertension and related complications. Obesity assessment includes body mass index (BMI), waist and hip circumferences, waist-to-hip ratio, mid-arm circumference, and skinfold thickness at various sites [6-8].

Anthropometric indicators of obesity are significantly associated with adverse lipid profiles [9]. Visceral fat contributes to dyslipidemia via the secretion of free fatty acids and pro-inflammatory cytokines. Understanding the relationship between anthropometric indices and lipid parameters may guide targeted interventions to reduce cardiometabolic risks [10,11].

Despite this link [9-13], few studies in India have explored these associations [14-17]. However, there exists a literature gap. For instance, the study by Reddy and Nambiar [17], although it included a much higher population of 306 adult females, the data pertained only to obese females. Similarly, the study by Panda et al. included a much higher sample size of 640 subjects, but it was a cross-sectional study without dividing the patients into hypertensives and controls. Moreover, the study was done in a peripheral region of India [16]. Therefore, the present study was conducted to assess various anthropometric indices and lipid profiles in hypertensive individuals, comparing them with normotensive controls. The results may allow to address the gap in the literature and widen the evidence of the relationship in a tertiary care center.

## Materials and methods

Study design

This research was conducted as a case-control study, which allowed comparison between participants with hypertension and participants without hypertension, to identify relevant associations.

Study location

The study was carried out in the Department of Medicine at Maharishi Markandeshwar Institute of Medical Sciences and Research (MMIMSR), Mullana, India, which provided the necessary clinical facilities and patient pool for the research.

Study duration

The study was conducted over a period of 18 months, beginning in January 2023 and concluding in June 2024, ensuring adequate time for patient recruitment, data collection, and analysis.

Patient selection

A total of 200 participants were enrolled in the study, comprising 100 participants with hypertension, who served as the case group, and 100 participants without hypertension, who served as the control group, selected based on predefined inclusion and exclusion criteria.

Inclusion criteria

The study included adult participants above 18 years of age who were diagnosed with essential hypertension and provided informed consent to participate. The control group comprised individuals without hypertension, either patients attending the outpatient department for non-hypertensive conditions or attendants of patients, who had no history or clinical evidence of hypertension.

Exclusion criteria

The study excluded patients with secondary causes of hypertension and pregnant women. Secondary causes of hypertension were systematically excluded through clinical evaluation and targeted investigations. A detailed history and physical examination were performed to rule out drug-induced or structural causes. Laboratory tests included renal function tests (serum creatinine, estimated glomerular filtration rate (eGFR), and urinalysis) to assess for renal parenchymal disease, along with serum electrolytes, particularly potassium, to evaluate for primary hyperaldosteronism. Endocrine causes were screened with thyroid function tests, plasma aldosterone-renin ratio, and, when clinically indicated, cortisol levels or metanephrines to exclude Cushing’s syndrome and pheochromocytoma. Additional evaluations, such as renal Doppler ultrasonography for renovascular disease and sleep studies for suspected obstructive sleep apnea, were undertaken where appropriate. Collectively, these assessments ensured exclusion of secondary hypertension before participant inclusion in the study. In addition, individuals who were unwilling to provide consent for participation and those already receiving lipid-lowering therapy were also excluded from the study.

Sample size

The sample size calculation was based on the study results of a previous study by Choudhury et al. [12], who observed that in hypertensives, mean ± SD of total cholesterol, triglyceride, and high-density lipoprotein (HDL), low-density lipoprotein (LDL) was 238.31 ± 3.39, 178.34 ± 6.31, 41.24 ± 3.22, and 151.28 ± 7.77, respectively, and in non-hypertensive group, it was 187.01 ± 6.25, 141.48 ± 11.29, 44.28 ± 5.63, and 110.31 ± 6.34, respectively. Taking these values as reference, the minimum required sample size with 95% power of study and 5% level of significance was 60 patients in each study group. To reduce margin of error, total sample size taken was 200 (100 patients per group).

For comparing mean of two groups, the following formula was used: \[N \geq \frac{2 (\text{SD})^2 \, (Z_{\alpha} + Z_{\beta})^2}{(\text{Mean Difference})^2}\]

Where Z_α_ is value of Z (normal variate) at two sided alpha error of 5% and Z_β_ is value of Z (normal variate) at power of 95%, and mean difference is difference in mean values of two groups.

Ethical clearance (IEC-2885 dated 20/3/2024) for conducting the study was obtained from Institutional Ethical Committee Maharishi Markandeshwar Institute of Medical Sciences and Research, Mullana, Ambala. After applying inclusion and exclusion criteria, 100 participants with hypertension and 100 participants without hypertension were included. Written informed consent was taken from both patients and controls.

Measurement

Data collection included age, gender, comorbidities, smoking, and alcohol status of the enrolled subjects. The physical examination findings were recorded (general, systemic, and vital assessment). For blood pressure (BP), all study assessments were conducted uniformly between 8:00 and 9:30 a.m. Following a five-minute period of rest, BP was recorded in the seated position from the left arm using a validated automated device (Omron HBP-1300: Omron Healthcare Co., Ltd., Kyoto, Japan). As part of quality assurance, the automated monitor was cross-checked against manual sphygmomanometer readings every 15 days throughout the study. For analysis, three consecutive readings were obtained, and the mean of the final two measurements was considered. The resulting average was rounded to the nearest integer. Participants with systolic BP ≥140 mmHg and/or diastolic BP ≥90 mmHg (in accordance with the Joint National Committee (JNC)-7 2017 diagnostic guidelines) or with the current use of antihypertensive medications were classified as “Participants with hypertension” [18]. Those with average readings below 140/90 mmHg were classified as “Participants without hypertension”. The absence of hypertension was confirmed by the study investigators through direct review of these standardized measurements. Further, the records of participants without hypertension were reviewed for any investigations in the last six months, to rule out secondary hypertension causes, and if tests were not available, further testing was not done.

Anthropometric measurements included waist and hip circumference, height, weight, and body mass index (BMI) (classified using Asia-Pacific guidelines) [13]. Waist-hip ratio (WHR = waist circumference (WC)/hip circumference (HC)) and waist-height ratio (WHtR = WC/height) were calculated. Skinfold thickness (SFT) was measured at four sites, biceps, triceps, subscapular, and suprailiac, using calipers and recorded in millimeters.

For biochemical assessment, 5 mL fasting venous blood was collected. Lipid profile (total cholesterol, triglycerides (TG), and high-density lipoprotein cholesterol (HDL-C)) was analyzed using the ADIVA Centaur automated system (Siemens Healthineers, Erlangen, Germany). Low-density lipoprotein cholesterol (LDL-C) was calculated using the Friedewald formula: LDL-C = total cholesterol (TC) - HDL-C - (TG/5); very low-density lipoprotein cholesterol (VLDL) as TG/5. Dyslipidemia was defined per National Cholesterol Education Program-Adult Treatment Panel (NCEP-ATP) III classification: TC ≥200 mg/dL, TG ≥150 mg/dL, and HDL-C <40 mg/dL (men) <50 mg/dL (women) [19]. Primary outcomes were the association between anthropometric indices and lipid profile with hypertension and their correlation.

Data analysis

Microsoft Excel (Microsoft Corporation, Redmond, Washington, USA) was utilized as the primary tool for initial data entry and storage. Patient information collected during the study was systematically entered into Excel spreadsheets, which allowed structured storage, easy accessibility, and preliminary validation of data. Each spreadsheet consisted of separate columns for demographic details (such as patient identification number, age, sex, and address), clinical variables, laboratory results, and anthropometry indices.

For analysis, variables were represented in tables and graphs as numbers with corresponding percentages, mean values with standard deviations, or median values with interquartile ranges (25th-75th percentile) and specified ranges. The Shapiro-Wilk test was applied to assess the normality of quantitative variables.

The Independent t-test was used to determine associations between quantitative variables, while the Chi-square test was applied for categorical (qualitative) variables. In cases where cell counts were less than expected, Fisher’s exact test was employed. Pearson’s correlation coefficient was used to evaluate the relationship between lipid profiles, anthropometric parameters, and skinfold thickness. All statistical analyses were performed using the Statistical Package for the Social Sciences (SPSS), version 25.0 (IBM Corp., Armonk, NY). A p-value of less than 0.05 was considered statistically significant.

## Results

The comparison of baseline characteristics showed that the mean age of participants with hypertension (59.47 ± 4.77 years) was slightly higher than that of participants without hypertension (58.64 ± 4.88 years), but the difference was not statistically significant (p = 0.225). Gender distribution was similar across groups, with 51% females and 49% males among participants with hypertension, compared to 45% females and 55% males among participants without hypertension (p = 0.396). The proportions of current/former smokers (8% vs. 5%, p = 0.390) and alcoholics (11% vs. 8%, p = 0.469) were also comparable between the two groups.

Most participants reported no comorbidity (97% vs. 99%), while a small proportion had chronic obstructive pulmonary disease (COPD) (3% vs. 1%), with no significant group differences (p = 0.621). As expected, systolic blood pressure was significantly higher among participants with hypertension (144.3 ± 11.02 mmHg) compared to participants without hypertension (120.06 ± 5.02 mmHg, p < 0.0001). Similarly, diastolic blood pressure was higher in participants with hypertension (93.78 ± 8.33 mmHg) compared to participants without hypertension (77.26 ± 4.48 mmHg, p < 0.0001) (Table 1).

**Table 1:** Comparison of demographic and clinical characteristics of study participants Values are reported in n (%) or *Mean ± SD. p < 0.05 was considered statistically significant. COPD: chronic obstructive pulmonary disease.

Parameter	Participants with hypertension	Participants without hypertension	p-value
Age (years)*	59.47 ± 4.77	58.64 ± 4.88	0.225
Gender: Female	51 (51%)	45 (45%)	0.396
Gender: Male	49 (49%)	55 (55%)	0.396
Current/former smokers	8 (8%)	5 (5%)	0.390
Alcoholics	11 (11%)	8 (8%)	0.469
No comorbidity	97 (97%)	99 (99%)	0.621
COPD	3 (3%)	1 (1%)	0.621
Systolic BP (mmHg)*	144.3 ± 11.02	120.06 ± 5.02	< 0.0001
Diastolic BP (mmHg)*	93.78 ± 8.33	77.26 ± 4.48	< 0.0001

Lipid profiles differed significantly between participants with hypertension and participants without hypertension. Participants with hypertension had significantly higher mean total cholesterol (200.8 ± 19.09 vs. 172.29 ± 14.1 mg/dL), mean triglycerides (147.48 ± 16.53 vs. 111.3 ± 12.04 mg/dL), mean LDL-C (131.79 ± 17.69 vs. 96.01 ± 12.67 mg/dL), and VLDL (29.49 ± 3.31 vs. 22.26 ± 2.41 mg/dL). HDL-C was significantly lower in participants with hypertension (39.52 ± 5.47 vs. 54.05 ± 5.95 mg/dL), with all differences statistically significant (p < 0.0001) (Table 2).

**Table 2:** Comparison of lipid profile of study participants Values are reported in Mean ± SD. Independent t test was applied for statistical analysis. p < 0.05 was considered for statistical significance. LDL: low-density lipoprotein, HDL: high-density lipoprotein, VLDL: very low-density lipoprotein.

Lipid parameter	Participants with hypertension (Mean ± SD)	Participants without hypertension (Mean ± SD)	p-value
Total cholesterol (mg/dL)	200.8 ± 19.09	172.29 ± 14.1	< 0.0001
Triglycerides (mg/dL)	147.48 ± 16.53	111.3 ± 12.04	< 0.0001
LDL (mg/dL)	131.79 ± 17.69	96.01 ± 12.67	< 0.0001
VLDL (mg/dL)	29.49 ± 3.31	22.26 ± 2.41	< 0.0001
HDL (mg/dL)	39.52 ± 5.47	54.05 ± 5.95	< 0.0001

Anthropometric comparisons showed no significant difference in height (p = 0.747), but weight, BMI, and weight-height ratio were significantly higher in participants with hypertension (p < 0.0001 for all). Waist circumference (102.62 ± 6.26 vs. 92.61 ± 3.05 cm), hip circumference (105.23 ± 4.7 vs. 100.21 ± 5.1 cm), waist-hip ratio (0.98 ± 0.08 vs. 0.93 ± 0.06), and mid-arm circumference (30.67 ± 2.12 vs. 27.54 ± 1.58 cm) were all significantly higher among participants with hypertension (p < 0.0001). Skinfold measurements at triceps, subscapular, and suprailiac sites were also significantly higher in participants with hypertension (p < 0.0001) (Table 3).

**Table 3:** Comparison of anthropometric indices of study participants Values are reported in Mean ± SD. Independent t test was applied for statistical analysis. p < 0.05 was considered for statistical significance.

Parameter	Participants with hypertension	Participants without hypertension	p-value
Height (cm)	163.13 ± 6.65	162.87 ± 4.54	0.747
Weight (kg)	72.33 ± 5.87	67.17 ± 4.22	< 0.0001
Body mass index (kg/m²)	27.32 ± 3.18	25.37 ± 2.06	< 0.0001
Weight-height ratio	0.44 ± 0.04	0.41 ± 0.03	< 0.0001
Waist circumference (cm)	102.62 ± 6.26	92.61 ± 3.05	< 0.0001
Hip circumference (cm)	105.23 ± 4.7	100.21 ± 5.1	< 0.0001
Waist-hip ratio	0.98 ± 0.08	0.93 ± 0.06	< 0.0001
Mid-arm circumference (cm)	30.67 ± 2.12	27.54 ± 1.58	< 0.0001
Biceps (mm)	7.47 ± 1.34	7.22 ± 1.93	0.288
Triceps (mm)	13.31 ± 1.55	10.95 ± 1.4	< 0.0001
Subscapular (mm)	25.37 ± 1.29	20.62 ± 2.18	< 0.0001
Suprailiac (mm)	24.02 ± 1.12	21.61 ± 0.89	< 0.0001

Correlations between anthropometric indices and lipid parameters (Tables 4, 5) indicated several noteworthy trends. Height demonstrated a positive association with HDL (r = 0.906, p < 0.0001). Weight showed positive relationships with total cholesterol (r = 0.95) and LDL (r = 1.00) (both p < 0.0001). BMI was positively associated with TC, TG, LDL, and VLDL (r = 0.221 to 0.722, all p < 0.05) and inversely related to HDL (r = -0.714, p < 0.0001). Similarly, the weight-height ratio showed positive associations with TC, TG, LDL, and VLDL, and a negative one with HDL (all p < 0.05). Waist circumference did not exhibit a significant correlation with lipid parameters, whereas hip circumference had a modest negative association with TC (r = -0.205, p = 0.041). Waist-hip ratio showed a positive association with HDL (r = 0.230, p = 0.021). Among skinfold thickness measures, triceps SFT was inversely related to TC (r = -0.212, p = 0.034) and LDL (r = -0.250, p = 0.012), while subscapular and suprailiac SFT demonstrated significant associations with TC and VLDL (p < 0.05).

**Table 4:** Correlation of anthropometric indices with lipid profile in participants with hypertension Test: Pearson correlation coefficient (r). p < 0.05 was considered for statistical significance. TC: total cholesterol, TG: triglycerides, LDL: low-density lipoprotein, HDL: high-density lipoprotein, VLDL: very low-density lipoprotein.

Parameter	TC	TG	HDL	LDL	VLDL
Height (m)	0.173 (p = 0.085)	-0.089 (p = 0.378)	0.906 (p < 0.0001)	-0.077 (p = 0.448)	-0.090 (p = 0.373)
Weight (kg)	0.950 (p < 0.0001)	0.191 (p = 0.057)	-0.038 (p = 0.710)	1.000 (p < 0.0001)	0.191 (p = 0.057)
BMI (kg/m²)	0.503 (p < 0.0001)	0.221 (p = 0.027)	-0.714 (p < 0.0001)	0.722 (p < 0.0001)	0.222 (p = 0.026)
Weight-height ratio	0.737 (p < 0.0001)	0.227 (p = 0.023)	-0.471 (p < 0.0001)	0.898 (p < 0.0001)	0.227 (p = 0.023)
Waist circumference (cm)	-0.067 (p = 0.508)	-0.136 (p = 0.177)	0.192 (p = 0.056)	-0.106 (p = 0.295)	-0.136 (p = 0.176)
Hip circumference (cm)	-0.205 (p = 0.041)	-0.152 (p = 0.131)	-0.131 (p = 0.195)	-0.152 (p = 0.131)	-0.152 (p = 0.132)
Waist-hip ratio (WHR)	0.045 (p = 0.658)	0.007 (p = 0.948)	0.230 (p = 0.021)	-0.023 (p = 0.817)	0.007 (p = 0.949)
Mid-arm circumference (cm)	-0.074 (p = 0.464)	0.039 (p = 0.703)	0.084 (p = 0.406)	-0.113 (p = 0.265)	0.038 (p = 0.708)

**Table 5:** Correlation of SFT with lipid profile in participants with hypertension Test: Pearson correlation coefficient (r). p < 0.05 was considered for statistical significance. TC: total cholesterol, TG: triglycerides, LDL: low-density lipoprotein, HDL: high-density lipoprotein, VLDL: very low-density lipoprotein, SFT: skin fold thickness.

Parameter	TC	TG	HDL	LDL	VLDL
Biceps SFT (mm)	-0.014 (p = 0.886)	-0.123 (p = 0.224)	0.090 (p = 0.372)	-0.021 (p = 0.835)	-0.124 (p = 0.218)
Triceps SFT (mm)	-0.212 (p = 0.034)	-0.116 (p = 0.251)	0.140 (p = 0.165)	-0.250 (p = 0.012)	-0.116 (p = 0.249)
Subscapular SFT (mm)	0.314 (p = 0.002)	0.994 (p < 0.0001)	-0.123 (p = 0.223)	0.191 (p = 0.056)	0.993 (p < 0.0001)
Suprailiac SFT (mm)	0.226 (p = 0.024)	-0.119 (p = 0.240)	0.997 (p < 0.0001)	-0.042 (p = 0.678)	-0.120 (p = 0.234)

## Discussion

The case-control study was meticulously designed to evaluate the association between anthropometric parameters, lipid profile abnormalities, and essential hypertension. A total of 200 individuals, comprising 100 participants with hypertension and 100 age- and sex-matched participants without hypertension, were included to ensure demographic parity and minimize confounding. We observed that all anthropometric indices (BMI, waist circumference, hip circumference, waist-hip ratio, and waist-height ratio) evaluated were significantly elevated in the participants with hypertension group (p-values < 0.0001).

In line with the present study, Kumar [20] documented significantly elevated BMI in participants with hypertension (26.15 ± 1.44 kg/m²) against participants without hypertension (24.34 ± 1.34 kg/m²; p < 0.01). Choudhury et al. [12] reported BMI values of 25.98 ± 3.39 and 23.58 ± 2.64 kg/m² in participants with hypertension and participants without hypertension, respectively (p = 0.001). Wu et al. [21] also found higher BMI among participants with hypertension (30.62 ± 7.05) than participants without hypertension (27.16 ± 6.12; p < 0.001).

Furthermore, triceps skinfold thicknesses were also significantly higher among participants with hypertension. Subscapular SFT averaged 25.37 ± 1.29 mm versus 20.62 ± 2.18 mm in participants without hypertension; suprailiac SFT was 24.02 ± 1.12 mm vs. 21.61 ± 0.89 mm; and triceps SFT was 13.31 ± 1.55 mm vs. 10.95 ± 1.40 mm (all p < 0.0001). In contrast, biceps SFT showed no significant difference between groups (7.47 ± 1.34 vs. 7.22 ± 1.93 mm; p = 0.288). This lack of difference may be explained by the poor sensitivity and reliability of biceps skinfold thickness compared to truncal and peripheral sites, as previously reported. The biceps site is more challenging to measure accurately due to its smaller absolute thickness and higher variability in compressibility, which makes it less responsive in detecting group differences. Additionally, prior work has emphasized that the reliability of skinfold measurements is strongly dependent on the evaluator’s experience, with the biceps site often showing poor reproducibility [22].

In comparison, Kumar [20] observed higher triceps (12.92 ± 1.66 vs. 10.93 ± 1.36 mm; p < 0.001), subscapular SFT (24.85 ± 1.50 vs. 21.53 ± 1.69 mm; p < 0.001), and biceps SFT (7.48 ± 1.37 vs. 7.15 ± 1.89 mm; p < 0.001) in participants with hypertension versus participants without hypertension. Vairamuthu and Thangavel [23] found that individuals with triceps and subscapular SFTs above the 95th percentile had significantly greater odds of hypertension.

The findings show that peripheral obesity in the form or biceps, triceps, or subscapular SFT may vary as per the fat distribution in different populations. The associations may depend on various other factors of diet, lifestyle, and exercise. However, it must be mentioned here that unlike peripheral fat, visceral fat has high metabolic activity, secreting pro-inflammatory cytokines, and may contribute more to dysregulation of insulin and lipids [22]. The non-significance of biceps SFT (especially in our study) shows the limited role of peripheral subcutaneous fat in hypertensive pathophysiology.

Furthermore, participants with hypertension demonstrated a distinctly atherogenic lipid profile with high cholesterol, TG, and low HDL. In corroboration, Choudhury et al. [12] reported similar findings where total cholesterol (238.31 ± 3.39 vs. 187.01 ± 6.25 mg/dL), LDL (151.28 ± 7.77 vs. 110.31 ± 6.34 mg/dL), and triglycerides (178.34 ± 6.31 vs. 141.48 ± 11.29 mg/dL) were higher and HDL was lower (41.24 ± 2.61 vs. 44.28 ± 2.15 mg/dL; all p = 0.001). These data affirm the close interplay between dyslipidemia and hypertension. This may be because visceral adiposity impairs lipid metabolism via increased hepatic VLDL synthesis, reduced HDL production, and delayed clearance of triglyceride-rich particles, all of which contribute to vascular injury and hypertension progression [12,17].

In terms of correlations, the analysis revealed that participants with hypertension exhibited significantly greater measures of general and central adiposity, along with a more atherogenic lipid profile. Waist-based indices, truncal skinfold thicknesses, and waist-to-height ratios demonstrated stronger correlations with dyslipidemia than BMI or peripheral skinfolds, suggesting that fat distribution, rather than overall body mass, holds greater metabolic and clinical significance. However, waist circumference showed no significant correlation with lipid parameters, which may be due to measurement variability from inconsistent landmarks, posture, or breathing, as well as homogeneity in BMI and adiposity within the sample. Additionally, alternative indices like waist-to-height ratio and BMI may have demonstrated stronger predictive value, thereby overshadowing waist circumference in explaining lipid profile associations. These findings reinforce the hypothesis that truncal adiposity exerts a more profound metabolic impact than peripheral fat [24,25]. Visceral fat depots modulate adipokine secretion, exacerbate insulin resistance, and impair lipid homeostasis, making them key contributors to cardiometabolic dysfunction [24-26].

Limitations

The cross-sectional study design could not establish causality between anthropometric indices, lipid profiles, and hypertension. The study was conducted at a single tertiary center, its findings may not generalize to diverse populations with different lifestyles. SFT, though standardized, is operator-dependent and subject to minor variability. The study did not control for key lifestyle factors such as diet, physical activity, or salt intake, which influence lipid levels and blood pressure. Other limitations included unadjusted antihypertensive medication use, diet, exercise, and lifestyle factors, possible bias in skinfold thickness measurement, implausibly high correlations, and missing data on renal disease, thyroid disorders, and other secondary causes of hypertension, reducing analytic validity. A limitation is that hypertension was defined using the JNC-7 cutoff of ≥140/90 mmHg, which is higher than the contemporary ≥130/80 mmHg threshold, and may have led to misclassification of some participants and potentially attenuated observed associations. Lastly, the potential impact of medications like antihypertensives or statins was not adjusted.

Future recommendations

Since the expected change in metabolic syndrome is an increase in triglycerides and decrease in HDL (secondary to decreased lipoprotein lipase activity at the adipose tissue and increased activity of cholesterol ester transfer protein, with an overproduction of VLDL by the liver, and reduced clearance), future studies are recommended to use the triglycerides:HDL ratio as a better variable to capture the effect of both.

## Conclusions

In conclusion, there was a significant association between central adiposity and adverse lipid profiles in individuals with essential hypertension. Hypertensive subjects showed significantly elevated waist-hip ratio, waist-height ratio, subscapular, and suprailiac skinfold thickness compared to normotensives (all p < 0.0001), while biceps skinfold showed no meaningful difference or lipid correlation. The lipid profile in hypertensives was distinctly atherogenic, with higher total cholesterol, LDL, VLDL, triglycerides, and lower HDL (all p < 0.0001). Subscapular SFT also strongly correlated with triglycerides (r = 0.994) and VLDL (r = 0.993). Based on the study results, it is recommended that waist-based and a combination of anthropometric measures be used over BMI, particularly for early risk detection in rural or resource-limited settings, offering a simple, cost-effective tool for hypertension-related dyslipidemia assessment.

## References

[1] Singh SD, Senff JR, van Duijn CM, Rosand J (2024). Treating hypertension: important for heart health, fundamental for brain health. Stroke.

[2] Harrison DG, Coffman TM, Wilcox CS (2021). Pathophysiology of hypertension: the Mosaic theory and beyond. Circ Res.

[3] Ma J, Chen X (2022). Advances in pathogenesis and treatment of essential hypertension. Front Cardiovasc Med.

[4] Zhang W, He K, Zhao H, Hu X, Yin C, Zhao X, Shi S (2021). Association of body mass index and waist circumference with high blood pressure in older adults. BMC Geriatr.

[5] Chen L, Zhang J, Zhou N, Weng JY, Bao ZY, Wu LD (2023). Association of different obesity patterns with hypertension in US male adults: a cross-sectional study. Sci Rep.

[6] Rhee EJ, Cho JH, Kwon H (2018). Association between abdominal obesity and increased risk for the development of hypertension regardless of physical activity: a nationwide population-based study. J Clin Hypertens (Greenwich).

[7] Çam HH, Ustuner Top F (2021). Prevalence of hypertension and its association with body mass index and waist circumference among adolescents in Turkey: a cross-sectional study. J Pediatr Nurs.

[8] Ren H, Guo Y, Wang D, Kang X, Yuan G (2023). Association of normal-weight central obesity with hypertension: a cross-sectional study from the China health and nutrition survey. BMC Cardiovasc Disord.

[9] Garg S, Vinutha S, Karthiyanee K, Nachal A (2012). Relation between anthropometric measurements and serum lipid profile among cardio-metabolically healthy subjects: a pilot study. Indian J Endocrinol Metab.

[10] Nimkarn N, Sewarit A, Pirojsakul K, Paksi W, Chantarogh S, Saisawat P, Tangnararatchakit K (2022). Waist-to-height-ratio is associated with sustained hypertension in children and adolescents with high office blood pressure. Front Cardiovasc Med.

[11] Cheng W, Wang L, Chen S (2022). Differences in lipid profiles and atherogenic indices between hypertensive and normotensive populations: a cross-sectional study of 11 Chinese cities. Front Cardiovasc Med.

[12] Choudhury KN, Mainuddin AK, Wahiduzzaman M, Islam SM (2014). Serum lipid profile and its association with hypertension in Bangladesh. Vasc Health Risk Manag.

[13] (2004). Appropriate body-mass index for Asian populations and its implications for policy and intervention strategies. Lancet.

[14] Kaur A, Madhukar M, Dhoat PS, Kaur N (2023). Anthropometric indices and its association with hypertension in Indian population: a study from tertiary care center of North India. J Pharm Bioallied Sci.

[15] Aruna R, Santhanakrishnan N, Raj JB (2024). Anthropometric indices as a predictive screening tool for hypertension among young Indian adults. Indian J Public Health.

[16] Panda PS, Jain KK, Soni GP, Gupta SA, Dixit S, Kumar J (2017). Prevalence of hypertension and its association with anthropometric parameters in adult population of Raipur city, Chhattisgarh, India. Int J Res Med Sci.

[17] Reddy RR, Nambiar S (2018). Correlation of anthropometric indices with lipid profile in adult females. Natl J Physiol Pharm Pharmacol.

[18] Choi WJ, Lee HS, Hong JH, Chang HJ, Lee JW (2019). Comparison of the JNC7 and 2017 American College of Cardiology/American Heart Association guidelines for the management of hypertension in Koreans: analysis of two independent nationwide population-based studies. Int J Environ Res Public Health.

[19] Lipsy RJ (2003). The National Cholesterol Education Program Adult Treatment Panel III guidelines. J Manag Care Pharm.

[20] Kumar A (2014). Correlation between anthropometric measurement, lipid profile, dietary vitamins, serum antioxidants, lipoprotein (a) and lipid peroxides in known cases of 345 elderly hypertensive South Asian aged 56-64 y: a hospital based study. Asian Pac J Trop Biomed.

[21] Wu LD, Kong CH, Shi Y, Zhang JX, Chen SL (2022). Associations between novel anthropometric measures and the prevalence of hypertension among 45,853 adults: a cross-sectional study. Front Cardiovasc Med.

[22] Machado DR, da Silva LS, Vaquero-Cristóbal R (2024). Reliability of skinfold measurements and body fat prediction depends on the rater's experience: a cross-sectional analysis comparing expert and novice anthropometrists [PREPRINT]. Res Sq.

[23] Vairamuthu GS, Thangavel A (2019). Body fat indices for identifying risk of hypertension in Indian children. Int J Contemp Pediatr.

[24] Jensen MD (2008). Role of body fat distribution and the metabolic complications of obesity. J Clin Endocrinol Metab.

[25] Lee MJ, Wu Y, Fried SK (2013). Adipose tissue heterogeneity: implication of depot differences in adipose tissue for obesity complications. Mol Aspects Med.

[26] Chait A, den Hartigh LJ (2020). Adipose tissue distribution, inflammation and its metabolic consequences, including diabetes and cardiovascular disease. Front Cardiovasc Med.

